# Prognostic impact of hepatorenal function in patients undergoing transcatheter tricuspid valve repair

**DOI:** 10.1038/s41598-021-93952-9

**Published:** 2021-07-13

**Authors:** Tetsu Tanaka, Refik Kavsur, Atsushi Sugiura, Johanna Vogelhuber, Can Öztürk, Marcel Weber, Vedat Tiyerili, Sebastian Zimmer, Georg Nickenig, Marc Ulrich Becher

**Affiliations:** Department of Internal Medicine II, Heart Center Bonn, University Hospital Bonn, Venusberg-Campus 1, 53127 Bonn, Germany

**Keywords:** Interventional cardiology, Cardiac device therapy

## Abstract

Hepatorenal dysfunction is a strong risk factor in patients with heart failure (HF). We investigated the prognostic significance of hepatorenal dysfunction in 172 consecutive patients undergoing transcatheter tricuspid valve repair (TTVR). The model for end-stage liver disease excluding international normalized ratio (MELD-XI) score was calculated as 5.11 × ln(serum total bilirubin [mg/dl]) + 11.76 × ln(serum creatinine [mg/dl]) + 9.44. Patients were stratified into two groups: high (≥ 14) or low (< 14) MELD-XI score, according to the best cut-off value to predict a one-year composite outcome consisting of all-cause mortality and HF hospitalization. Compared to patients with low MELD-XI score (n = 121), patients with high MELD-XI score (n = 51) had a higher incidence of the composite outcome (47.1% vs. 17.4%; *p* < 0.0001). In the multivariable analysis, the MELD-XI score was an independent predictor of the composite outcome (adjusted hazard ratio: 1.12; 95% confidence interval [CI] 1.05–1.19; *p* = 0.0003). In addition, post-procedural TR < 3 + after TTVR was independently associated with a reduction in MELD-XI score six months after TTVR (adjusted odds ratio: 3.37; 95% CI 1.09–10.40; *p* = 0.03). Thus, the MELD-XI score was associated with the risk of one-year composite outcome, consisting of mortality and HF hospitalization, after TTVR and may help the risk stratification in patients undergoing TTVR.

## Introduction

Tricuspid regurgitation (TR) is a common valvular disease in patients with heart failure (HF) and is associated with impaired functional capacity and reduced long-term survival^1–3^. The treatment options for TR are still limited, and surgical repair for isolated TR is still controversial because of the high risk associated with the surgery^4,5^. TR patients often have multiple comorbidities that contribute to an elevated surgical risk. Therefore, minimally invasive catheter-based procedures are thought to be a promising alternative to reduce TR with lower procedural risks^6,7^. Recently, encouraging results with several different approaches of transcatheter tricuspid valve repair (TTVR) have been reported^8–11^. Although TTVR is alternative to surgical TV repair with a comparably low level of invasiveness, approximately 40% of the TTVR patients still experience adverse clinical events, including mortality or hospitalization due to heart failure^12^. Data regarding the characteristics of patients who are more likely to benefit from such interventions are still lacking. Thus, it is crucial to identify simple and useful tools for risk stratification, to support clinical decision-making in TTVR.

Multi-organ dysfunction, including hepatic and renal dysfunction, affects the prognosis and complicates the management of heart failure^13–15^. Cardiac dysfunction can lead to the renal or hepatic dysfunction and vice versa, the so-called cardiorenal and cardiohepatic syndromes. TR is associated with right-heart venous congestion and reduced forward stroke volume^16,17^, which contributes to hepatorenal dysfunction. The presence of hepatorenal dysfunction is associated with impaired clinical prognosis in patients with HF and TR^18–20^. However, the prognostic value of hepatorenal dysfunction on TTVR has not been well studied.

The Model for End-stage Liver Disease eXcluding International normalized (MELD-XI) score is one of the scoring models that have been widely used for the assessment of renal and hepatic function^21^. The MELD-XI score reflects liver and renal function and is calculated based on serum total bilirubin and creatinine levels. Previous reports have shown the prognostically predictive value of the MELD-XI score in patients with HF^19,20,22,23^. In the present study, we investigated the association between the MELD-XI score and clinical outcome after TTVR.

## Results

### Clinical characteristics of the study population

Of 195 patients who underwent their first TTVR, 23 patients were excluded from the present analysis, including 4 patients with hemodialysis, 11 patients with concomitant transcatheter mitral valve repair, and 8 patients without sufficient laboratory data for assessments of the MELD-XI score. Consecutively, a total of 172 patients were analyzed. The mean age of the patients was 77.3 ± 7.3 years and 39% were of male gender (Table 1). Eighty-seven percent of the patients were in the New York Heart Association (NYHA) functional class III or IV. The expected risk for surgical mortality was elevated, 15.1% [IQR 8.2% to 26.6%], as assessed by the median logistic European System for Cardiac Operative Risk Evaluation (EuroSCORE). The pre-procedural TR severity was graded as 3 + , 4 + , or 5 + in 49%, 40% and 11% of the study participants, respectively (Table 2). The mean left ventricular ejection fraction (LVEF) was 55.6 ± 10.2%, the mean tricuspid annular plane systolic excursion (TAPSE) was 17.7 ± 5.2 mm, and the secondary etiology of TR was observed in 96% of the patients.

**Table 1:** Baseline characteristics.

	Total	High MELD-XI (≥ 14)	Low MELD-XI (< 14)	*p* value
	n = 172	n = 51	n = 121	
Age (years)	77.3 ± 7.3	79.1 ± 6.3	76.6 ± 7.5	0.04
Male, n (%)	67 (39.0)	27 (52.9)	40 (33.1)	0.02
BMI (kg/m^2^)	26.0 ± 5.2	26.1 ± 5.0	26.0 ± 5.3	0.89
Diabetes, n (%)	48 (27.9)	17 (33.3)	31 (25.6)	0.30
Hypertension, n (%)	149 (86.6)	46 (90.2)	103 (85.1)	0.37
CAD, n (%)	95 (55.2)	32 (62.8)	63 (52.1)	0.20
Prior CABG, n (%)	41 (23.8)	14 (27.5)	27 (22.3)	0.47
Prior valve intervention, n (%)	68 (39.5)	22 (43.1)	46 (38.0)	0.53
Previous myocardial infarction, n (%)	46 (26.7)	11 (21.6)	35 (28.9)	0.32
Previous stroke, n (%)	24 (14.0)	8 (15.7)	16 (13.2)	0.67
Atrial fibrillation, n (%)	159 (92.4)	48 (94.1)	111 (91.7)	0.69
**NYHA functional class**				0.99
II, n (%)	23 (13.4)	7 (13.7)	16(13.2)	
III, n (%)	118 (68.6)	35 (68.6)	83 (68.6)	
IV, n (%)	31 (18.0)	9 (17.7)	22 (18.2)	
Lead across tricuspid valve, n (%)	55 (32.0)	18 (35.3)	37 (30.6)	0.54
COPD, n (%)	40 (23.3)	12 (23.5)	28 (23.1)	0.96
Logistic EuroSCORE (%)	15.1 [8.2, 26.6]	17.9 [8.9, 30.9]	14.7 [7.9, 23.7]	0.20
NT-pro BNP (pg/ml)	1838 [1006, 3726]	3264 [2153, 7185]	1584 [906, 2561]	< 0.0001
**Medication at baseline**				
Beta-blocker, n (%)	147 (85.5)	46 (90.2)	101 (83.5)	0.24
RAS inhibitor, n (%)	113 (65.7)	30 (58.8)	83 (68.6)	0.36
Loop diuretics, n (%)	162 (94.2)	49 (96.1)	113 (93.4)	0.27
Standardized furosemide equivalent (mg/day)	40 [20, 80]	40 [20, 150]	30 [20, 80]	0.01
Aldosterone antagonist, n (%)	79 (45.9)	25 (49.0)	54 (42.1)	0.64

Values shown are either n (%), mean ± SD, or median [interquartile range]. MELD-XI score Model for End-stage Liver Disease eXcluding International normalized score, BMI body mass index, CAD coronary artery disease, CABG coronary artery bypass grafting, NYHA New York Heart Association, COPD chronic obstructive pulmonary disease, NT-proBNP N-terminal pro-B-type natriuretic peptide, EuroSCORE European System for Cardiac Operative Risk Evaluation, RAS renin angiotensin system.

**Table 2:** Echocardiographic findings.

	Total	High MELD-XI (≥ 14)	Low MELD-XI (< 14)	*p* value
	n = 172	n = 51	n = 121	
**Etiology of TR**				0.43
Primary TR, n (%)	7 (4.1)	3 (5.9)	4 (3.3)	
Secondary TR, n (%)	165 (95.9)	48 (94.1)	117 (96.7)	
**TR severity**				0.64
3 + , n (%)	85 (49.4)	25 (49.0)	60 (49.6)	
4 + , n (%)	69 (40.1)	19 (37.3)	50 (41.3)	
5 + , n (%)	18 (10.5)	7 (13.7)	11 (9.1)	
**TR jet location**				
Central or Antero-septal commissure, n (%)	171 (99.4)	51 (100.0)	120 (99.2)	1.00
Postero-septal commissure, n (%)	108 (62.8)	31 (60.8)	77 (63.6)	0.73
Antero-posterior commissure, n (%)	63 (36.6)	17 (33.3)	46 (38.0)	0.56
Vena contracta (mm)	12.1 [9.3, 16.0]	11.0 [8.2, 15.1]	12.3 [9.8, 16.0]	0.20
EROA (mm^2^)	50 [37, 71]	42 [31, 59]	53 [40, 80]	0.006
LVEF (%)	55.6 ± 10.2	52.9 ± 12.5	56.8 ± 8.9	0.02
LVESV (ml)	29 [19, 41]	35 [20, 53]	28 [18, 37]	0.02
LVEDV (ml)	68 [47, 95]	74 [31, 121]	67 [44, 90]	0.02
RA area (cm^2^)	34.1 ± 11.4	35.6 ± 13.3	33.4 ± 10.4	0.26
RV diameter (mm)	51.9 ± 9.2	53.4 ± 9.8	51.3 ± 8.9	0.20
SPAP (mmHg)	36 ± 15	36 ± 14	36 ± 15	0.93
TAPSE (mm)	17.7 ± 5.2	17.0 ± 4.8	18.0 ± 5.3	0.24

Values shown are either n (%), mean ± SD, or median [interquartile range].

Legends: MELD-XI score = Model for End-stage Liver Disease eXcluding International normalized score; TR = tricuspid regurgitation; EROA = effective regurgitant orifice area; LVEF = left ventricular ejection fraction; LVEDV = left ventricular end-diastolic volume; LVESV = left ventricular end-systolic volume; RA = right atrium; RV = right ventricle; SPAP = systolic pulmonary artery pressure; TAPSE = tricuspid annular plane systolic excursion.

Procedural success was achieved in 156 patients (91%) (Table 3). Edge-to-edge repair was performed in 81% of patients with the MitraClip/TriClip or PASCAL systems, whereas annuloplasty was performed in 18% with the Cardioband or Trialign systems. One patient was simultaneously treated with PASCAL and Cardioband systems.

**Table 3:** Periprocedural findings and clinical outcomes after TMVR.

	Total	High MELD-XI (≥ 14)	Low MELD-XI (< 14)	*p* value
	n = 172	n = 51	n = 121	
**Procedural findings**				
**Device type**				0.36
Edge-to-edge repair, n (%)	140 (81.4)	43 (84.3)	97 (80.2)	
MitraClip/TriClip, n (%)	110 (64.0)	36 (70.6)	74 (61.2)	
PASCAL, n (%)	30 (17.4)	7 (13.7)	23 (19.0)	
Annuloplasty, n (%)	31 (18.0)	7 (13.7)	24 (19.8)	
Cardioband, n (%)	28 (16.3)	6 (11.8)	22 (18.2)	
Trilign, n (%)	3 (1.7)	2 (3.9)	1 (0.8)	
Combined therapy, n (%)	1 (0.6)	0 (0.0)	1 (0.8)	
Device success, n (%)	160 (93.0)	46 (90.2)	114 (94.2)	0.18
Number of Clips	1.8 ± 0.8	1.8 ± 0.9	1.8 ± 0.8	0.94
**Postprocedural echocardiographic findings**				
**Post TR grade**				0.71
0 or 1 + , n (%)	33 (19.1)	6 (11.8)	27 (22.3)	
2 + , n (%)	80 (46.5)	26 (51.0)	54 (44.6)	
3 + , n (%)	51 (29.7)	16 (31.4)	35 (28.9)	
4 + , n (%)	5 (2.9)	2 (3.9)	3 (2.5)	
5 + , n (%)	3 (1.7)	1 (2.0)	2 (1.7)	
Mean reduction in TR grade (grade)	1.4 ± 0.8	1.3 ± 0.7	1.4 ± 0.8	0.34
TR reduction of grade one at least, n (%)	156 (90.7)	46 (90.2)	110 (90.9)	0.88
Post mean TVPG (mmHg)	2.3 [1.6, 3.3]	2.7 [1.7, 3.9]	2.1 [1.6, 2.9]	0.23
**Postprocedural complications**				
Major or Life-threatening bleeding, n (%)	14 (8.1)	4 (7.8)	10 (8.3)	0.93
Acute kidney injury, n (%)	19 (11.1)	10 (19.6)	9 (7.4)	0.02
**Clinical outcomes**				
Composite outcome, n (%)	45 (26.2)	24 (47.1)	21 (17.4)	< 0.0001
All-cause death, n (%)	22 (12.8)	13 (25.5)	9 (7.4)	0.001
Cardiovascular death, n (%)	14 (8.1)	7 (13.7)	7 (5.8)	0.08
HF hospitalization, n (%)	32 (18.6)	17 (33.3)	15 (12.4)	0.001

Values shown are either n (%), mean ± SD, or median [interquartile range].MELD-XI score Model for End-stage Liver Disease eXcluding International normalized score, TR tricuspid regurgitation, TVPG tricuspid valve pressure gradient, HF heart failure.

### Analysis of MELD-XI score

**Figure 1:**
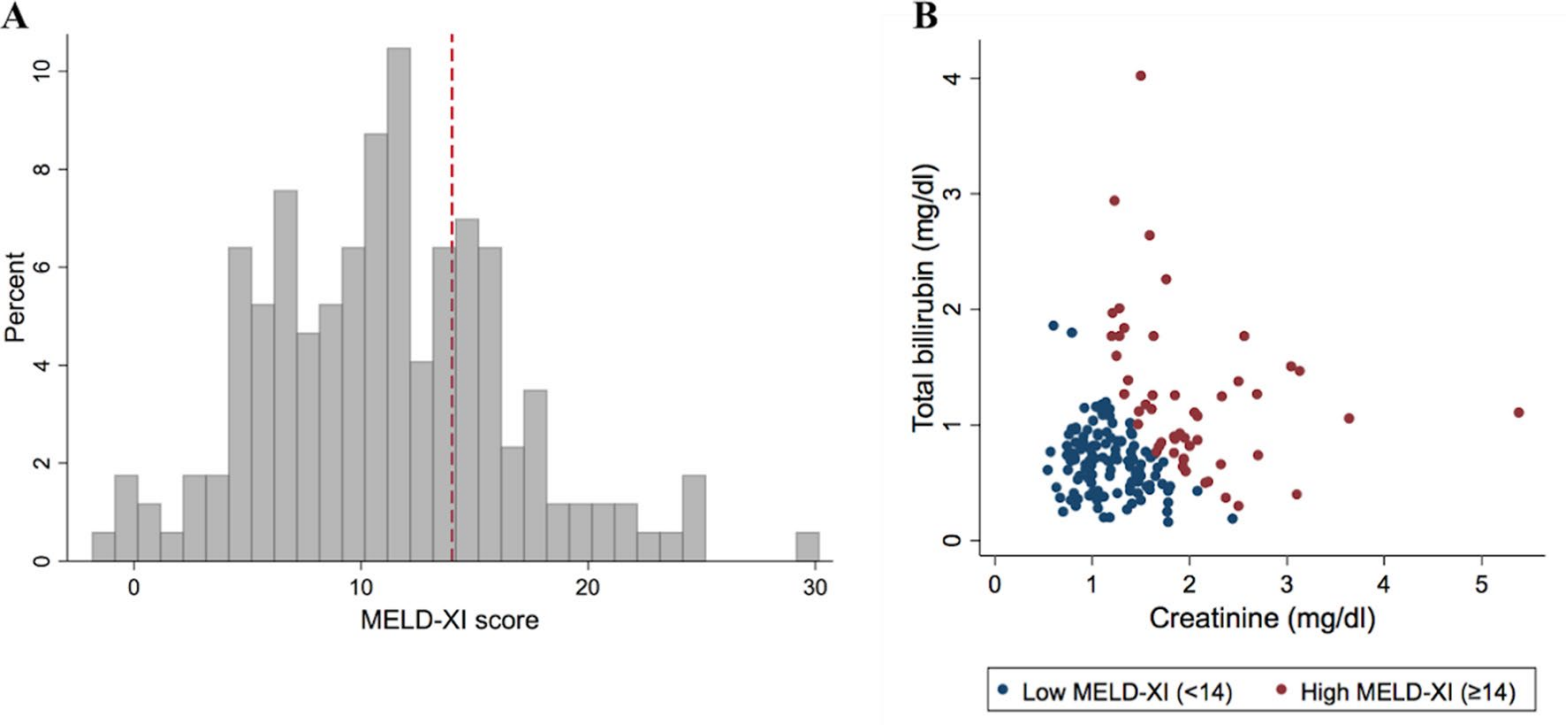
Distribution of the MELD-XI score and the components. The distribution of the MELD-XI score (**A**) and the creatinine and total bilirubin levels that are components of the MELD-XI score (**B**). The dashed red line indicates a MELD-XI score of 14.

The mean MELD-XI score was 11.0 ± 5.5 (Table 4), and the distribution of the MELD-XI score and its components, total bilirubin and creatinine levels, are presented in Fig. 1. In addition, the mean estimated glomerular filtration rate (eGFR) was 48.7 ± 22.2 ml/min/1.73 m^2^, and the mean total bilirubin was 0.74 mg/dl [IQR 0.53 mg/dl to 1.00 mg/dl]. The MELD-XI score was correlated to age, sex, LVEF, LV end-diastolic volume, and right ventricular dimension (Supplemental Table 1). In the multivariable analysis, age had the strongest correlation (standardized β: 0.32; 95% CI 0.17–0.46; *p* < 0.0001), followed by LVEF (standardized β: − 0.29; 95% CI − 0.49 to − 0.17; *p* = 0.0007).

**Table 4:** Assessment of hepatorenal function at baseline.

	Total	High MELD-XI (≥ 14)	Low MELD-XI (< 14)	*p* value
	n = 172	n = 51	n = 121	
MELD-XI score	11.0 ± 5.5	17.4 ± 3.5	8.4 ± 3.6	< 0.0001
Creatinine (mg/dl)	1.29 [0.99, 1.67]	1.85 [1.55, 2.32]	1.11 [0.92, 1.41]	< 0.0001
Total bilirubin (mg/dl)	0.74 [0.53, 1.00]	1.11 [0.77, 1.51]	0.69 [0.47, 0.85]	< 0.0001
eGFR (ml/min/1.73m^2^)	48.7 ± 22.2	31.6 ± 10.8	55.9 ± 21.8	< 0.0001

Values shown are either mean ± SD or median [interquartile range]. MELD-XI score Model for End-stage Liver Disease eXcluding International normalized score, eGFR estimated glomerular filtration rate.

Within one year after TTVR, 45 patients (26.2%) experienced the composite outcome, including 22 patients (12.8%) who died, 14 patients (8.1%) who died due to cardiovascular causes, and 32 patients (18.6%) who were re-hospitalized due to worsening HF (Table 3). The ROC analysis showed that the best MELD-XI score to discern the one-year composite outcome was 14 (Supplemental Figure 1). The Harrel’s C-statistic of MELD-XI score for the one-year composite outcome was 0.68 (95% CI 0.60–0.77; *p* < 0.0001), and those of eGFR and total bilirubin were 0.66 (95% CI 0.58–0.74; *p* < 0.0001) and 0.56 (95% CI 0.58–0.74; *p* < 0.0001), respectively (Supplemental Table 2).

According to the cut-off value, 51 patients (29.7%) had a high MELD-XI score (≥ 14). Patient characteristics and echocardiographic findings for each group are shown in Tables 1 and 2. Patients with a high MELD-XI score (≥ 14) were older and a higher proportion were male than those with a low MELD-XI score (< 14) (79.1 ± 6.3 years vs. 76.6 ± 7.5 years; *p* = 0.04, and 52.9% vs. 33.1%; *p* = 0.02, respectively). LVEF was lower in patients with a high MELD-XI score (≥ 14) than those with a low MELD-XI score (< 14) (52.9 ± 12.5% vs. 56.8 ± 8.9%; *p* = 0.02), whereas TAPSE, TR severity, and procedural findings were comparable between the two groups.

### Association between the MELD-XI score and clinical outcome after TTVR

Device type, post-procedural echocardiographic findings, and the rate of procedural success were comparable between the high MELD-XI score (≥ 14) and low MELD-XI score (< 14) groups. The incidence of AKI after TTVR was higher in patients with a high MELD-XI score (≥ 14) than those with low MELD-XI score (< 14) (19.6% vs. 7.4%; *p* = 0.02).

**Figure 2:**
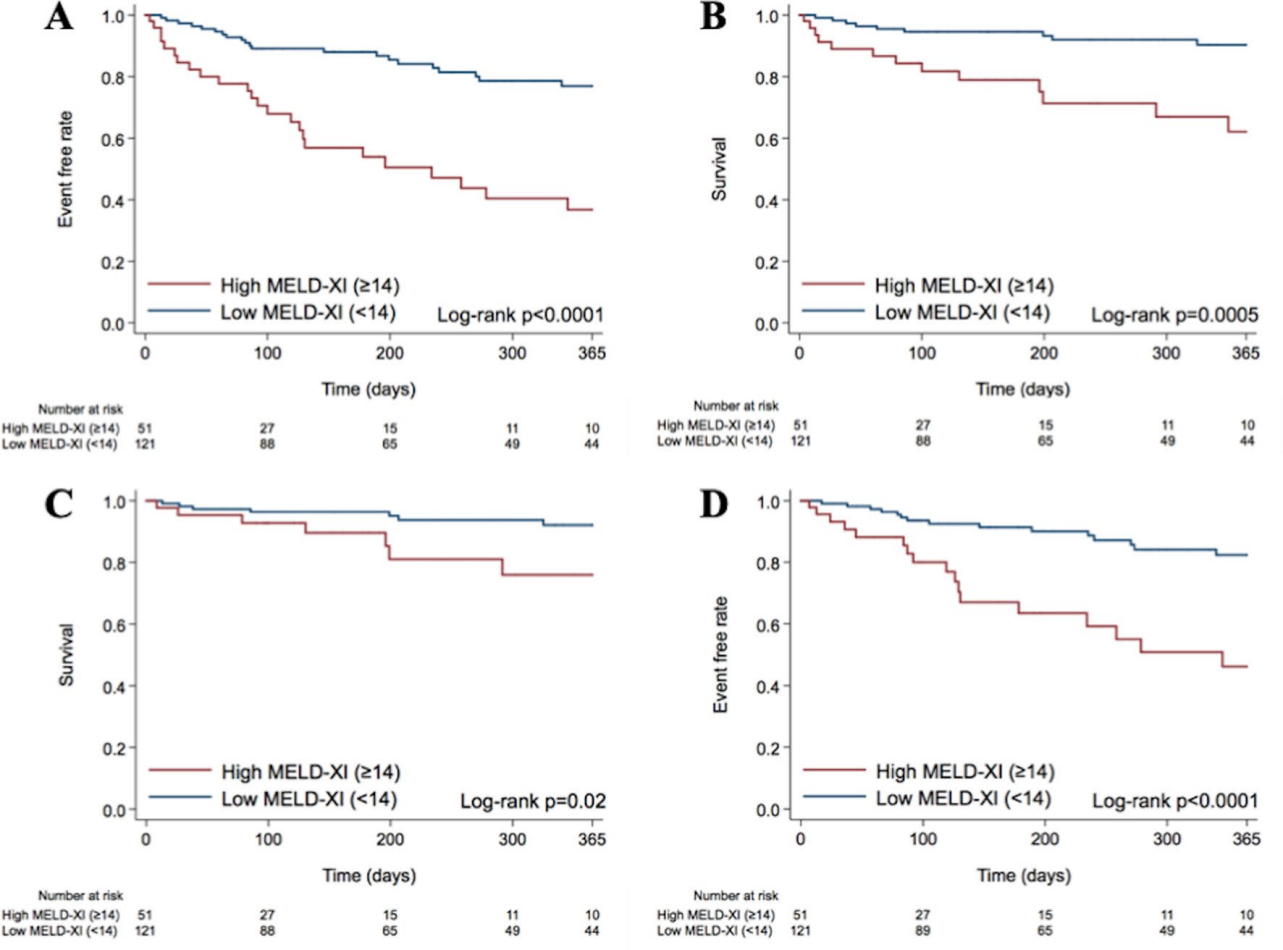
Clinical outcome according to the MELD-XI score within one year after TTVR. Kaplan–Meier curves demonstrating clinical outcomes within one year after TTVR, including the composite outcome (**A**), all-cause mortality (**B**), cardiovascular mortality (**C**), and HF hospitalization (**D**), according to the MELD-XI score.

Patients with a high MELD-XI score (≥ 14) had a higher incidence of the one-year composite outcome compared to those with a low MELD-XI score (< 14) (47.1% vs. 17.4%; *p* < 0.0001). Moreover, the incidences of all-cause mortality and HF hospitalization were higher in patients with a high MELD-XI score (≥ 14) than in those with a low MELD-XI score (< 14) (25.5% vs. 7.4%; *p* = 0.001, and 33.3% vs. 12.4%; *p* = 0.001, respectively). The incidence of cardiovascular mortality was numerically higher in patients with a high MELD-XI score (≥ 14) than in those with a low MELD-XI score (< 14) (13.7% vs. 5.8%; *p* = 0.08). The Kaplan–Meier curves for each of the clinical outcomes are shown in Fig. 2.

The MELD-XI score was associated with an incidence of the one-year composite outcome after TTVR (HR 1.12; 95% CI 1.07–1.20; *p* < 0.0001) (Table 5 and Supplemental Table 3). In a multivariable analysis, the MELD-XI score as a continuous variable remained an independent predictor of the composite outcome (adjusted HR in model 1: 1.13; 95% CI 1.05–1.19; *p* = 0.0003, and adjusted HR in model 2: 1.13; 95% CI 1.06–1.20; *p* = 0.0001). In addition, a high MELD-XI score (≥ 14) was also an independent predictor of the composite outcome (adjusted HR in model 1: 3.39; 95% CI 1.83–6.29; *p* = 0.0001, and adjusted HR in model 2: 3.83; 95% CI 2.07–7.13; *p* < 0.0001).

**Table 5:** Multivariable analysis for association of the MELD-XI score with an incidence of the composite outcome within two years after TMVR.

	Univariate analysis			Multivariable analysis		
	HR	95% CI	*p* value	HR	95% CI	*p* value
**Model 1: Clinical parameters**						
MELD-XI score*	1.13	1.07—1.20	< 0.0001	1.12	1.05—1.19	0.0003
High MELD-XI score (≥ 14)*	3.83	2.13—6.95	< 0.0001	3.39	1.83—6.29	0.0001
CAD	1.89	1.03—3.61	0.04	1.47	0.75—2.98	0.26
Logistic EuroSCORE (%)	1.02	1.00—1.04	0.01	1.02	0.99—1.04	0.07
NYHA class IV	2.11	1.09—3.88	0.03	2.58	1.27—4.99	0.01
**Model 2: Echocardiographic parameters**						
MELD-XI score*	1.13	1.07—1.20	< 0.0001	1.13	1.06—1.20	0.0001
High MELD-XI score (≥ 14)*	3.83	2.13—6.95	< 0.0001	3.83	2.07—7.13	< 0.0001
LVEF per 10% increase	0.72	0.56—0.94	0.01	0.97	0.73—1.30	0.82
TAPSE (mm)	0.90	0.84—0.96	0.003	0.91	0.84—0.97	0.01
TR reduction of at least one grade	0.36	0.18—0.84	0.02	0.24	0.11—0.57	0.002

* included separately in the multivariable analysis.

Legends: MELD-XI score = Model for End-stage Liver Disease eXcluding International normalized score, CAD = coronary artery disease; EuroSCORE = European System for Cardiac Operative Risk Evaluation; NYHA = New York Heart Association; LVEF = left ventricular ejection fraction; TAPSE = tricuspid annular plane systolic excursion; TR = tricuspid regurgitation.

### Improvement of MELD-XI score after TTVR

In 62 patients who at six months after TTVR had available total bilirubin and creatinine levels, the serial changes in the MELD-XI score were evaluated. The mean MELD-XI score at the follow-up was 11.0 ± 7.4, and 26 patients (64.2%) had a reduction in the MELD-XI score (Supplemental Table 4). In a multivariable logistic regression analysis, post-procedural TR < 3 + was associated with a MELD-XI reduction at six-month follow-up (adjusted OR: 3.37; 95% CI 1.09–10.40; *p* = 0.03), while baseline LV end-diastolic volume was inversely related to a MELD-XI reduction (adjusted OR for a 10 ml increase: 0.98; 95% CI 0.95–0.99; *p* = 0.03) (Supplemental Table 5).

## Discussion

This is the first study assessing the prognostic impact of hepatorenal function in HF-patients undergoing TTVR due to advanced TR. The main findings are summarized as follows: (1) the MELD-XI score, as both a continuous and dichotomous variable, was independently associated with the risk of the composite outcome of mortality and HF hospitalization within one year after TTVR; and (2) a sufficient TR reduction by TTVR with post-procedural TR < 3 + was independently associated with an improvement in MELD-XI score at six-month follow-up.

Hepatic and renal dysfunctions are associated with a higher surgical risk for TR. Accordingly, the current guidelines recommend TV interventions for severe symptomatic isolated TR before the onset of multi-organ damage to the liver and kidney^24^. However, the appropriate tool for pre-procedural assessment of multi-organ damage has not yet been identified. Since both renal and hepatic dysfunction are strong predictors of adverse clinical outcomes in patients with HF^13,15^, the combination of both can increase their ability for risk stratification. The MELD-XI score is one of the most established scoring systems for hepatorenal dysfunction, which is an alternative to the MELD scoring system that excludes the international normalized ratio from the calculation. The predictive value of the score has recently been proven in patients with HF^20,22,23^. In patients undergoing surgical tricuspid valve repair, the association of the MELD-XI score with the incidence of adverse events has been reported in previous studies^20,25^. In the present study, we revealed the association of the MELD-XI score, as both a dichotomous and continuous variable, with the risk of adverse events within one year after TTVR, such as all-cause mortality, cardiovascular mortality, and HF hospitalization. Additionally, patients with a high MELD-XI score had a higher incidence of AKI during the hospitalization for TTVR. These findings underline the prognostic impact of hepatorenal function and the utility of the MELD-XI score as a simple tool for risk stratification in patients undergoing TTVR.

Hepatorenal function can reflect altered hemodynamics and thus, improve after TV interventions^20,25^. Improvement of the MELD-XI score after surgical tricuspid valve repair was associated with better prognosis. Reduction of the regurgitant volume load by the TV interventions can relieve venous congestion and multi-organ hypoperfusion and improve hepatorenal function. In contrast, an insufficient reduction of TR can prolong venous congestion and hepatorenal hypoperfusion, which can lead to a further progression of hepatorenal dysfunction. In the present study, a reduction in the MELD-XI score was observed in approximately 40% of the patients who underwent serial assessments for 6-months follow-up after TTVR. Post-procedural TR of less than 3 + upon discharge was an independent predictor of a reduction in the MELD-XI score, while baseline LV end-diastolic volume was inversely related to MELD-XI improvement. These findings are in accordance with the previous studies in surgical TV repair^25^. Thus, it might be necessary, especially for patients with renal or hepatic dysfunction, to achieve a sufficient reduction of TR by TTVR for the sake of improving their hepatorenal function.

Recently, the post-procedural changes in multi-organ function, including hepatorenal function, after TTVR has attracted attention amongst clinician scientists. Karam et al. showed significant reductions in total bilirubin and aspartate transaminase levels at six months after TTVR, while the renal function did not significantly change^26^. However, the number of patients in the study who were evaluated at the follow-up was small, and patients with concomitant transcatheter mitral valve repair were also included. Besler et al. found that nutritional status improved after TTVR in patients with symptomatic TR^27^. Malnutrition is one of the multi-organ damages that are attributed to venous congestion and hypoperfusion due to TR as well as hepatorenal dysfunction. Furthermore, a reduction in TR by TTVR was a predictor of the improvement in nutritional status, which was associated with a better outcome. Our findings were in agreement with previous reports. However, the underlying mechanisms of the improvement in hepatorenal function after TTVR are still insufficiently understood. Larger studies are needed to assess predictors of the improvement in hepatorenal function after TTVR.

There are several limitations to this study that should be acknowledged. Although the present study included a comparably high number of patients for this novel field, the single-centric and observational characteristics of the present study might have impacted our results owing to patient selection bias. Furthermore, the possibility remains that confounders were insufficiently considered in the multivariable analysis. Nevertheless, we conducted several multivariable models, adjusting for various clinical or echocardiographic covariates, which may at least partially address the issue. Second, we could not distinguish acute hepatorenal injury from chronic dysfunction, because we only evaluated the MELD-XI score from the latest laboratory data before TTVR. Finally, we evaluated serial measurements of the MELD-XI score after TTVR. However, approximately 60% of the patients could not be evaluated because of death within six months or a lack of the laboratory data at the follow-up.

In conclusion, hepatorenal dysfunction, represented by the MELD-XI score, was independently associated with the composite outcome of all-cause mortality and hospitalization due to heart failure after TTVR, irrespective of other clinically important variables such as LVEF, TAPSE, and coronary artery disease. Moreover, HF patients with a high MELD-XI score (≥ 14) had a higher incidence of all-cause mortality and HF hospitalization within one year after TTVR and had higher rates of post-procedural AKI. Post-procedural TR < 3 + was associated with a reduction in the MELD-XI score within the six-month follow-up period. The MELD-XI score is a simple and objective scoring system to assess renal and hepatic functions, which are both aggravated by the progression of TR. Therefore, the MELD-XI score can aid in pre-procedural risk-stratification, patient selection, and decision-making for the timing of TTVR in patients with TR.

## Methods

### Study population

This study was a retrospective analysis of data from a local registry, which is a single-center, prospective, consecutive database of patients treated at the University of Bonn^28,29^. We analyzed consecutive patients who underwent TTVR from June 2015 to September 2020 and had available the pre-procedural serum creatinine and total bilirubin results. We excluded patients with hemodialysis and those who underwent concomitant transcatheter mitral valve repair.

### Procedure

The indication for TTVR was severe or greater TR accompanied by symptomatic HF according to the NYHA functional classification in patients considered as inoperable or at a high surgical risk. After a standardized diagnostic workup, including transesophageal echocardiography (TEE), the decision to perform the intervention was taken by the interdisciplinary heart team. Procedures were performed using the MitraClip/TriClip (Abbott Vascular, Santa Clara, California), PASCAL (Edwards Lifesciences, Irvine, California), Cardioband (Edwards Lifesciences), or Trialign (Edwards Lifesciences) devices under general anesthesia with three-dimensional TEE and fluoroscopic guidance. Details of the device system and procedure have previously been well described^8,10,30,31^. The discretion of whether a second or third device needed to be used was left up to the treating physicians. Procedural success was defined as successful device implantation and a reduction of TR by ≥ 1 grade, as assessed by transthoracic echocardiography upon discharge after TTVR.

### Assessment of hepatorenal function

The hepatorenal function was assessed using the MELD-XI score, which was calculated as 5.11 × ln(serum total bilirubin in mg/dl) + 11.76 × ln(serum creatinine in mg/dl) + 9.44^21^. Serum creatinine and total bilirubin results that were taken within the one week prior to TTVR were included. In addition, postprocedural measurements of these values were collected at six months after TTVR, and a reduction in MELD-XI score was defined as: baseline MELD-XI score was higher than post-procedural MELD-XI score at follow-up. Acute kidney injury (AKI) was defined according to the Acute Kidney Injury Network criteria as an absolute increase in serum creatinine of ≥ 0.3 mg/dl or a relative increase of ≥ 50% from baseline to 48 h after the procedure^32^.

### Echocardiographic parameters

We assessed echocardiographic parameters that were collected at baseline and discharge, according to the current guidelines^33^. TEE was performed at baseline and during the procedure with a Vivid E95 ultrasound system (GE Healthcare, Illinois, USA). According to a combination of semiquantitative and quantitative assessments, the severity of TR was graded as follows: grade 0, none; 1 + , mild; 2 + , moderate; 3 + , severe; 4 + , massive; 5 + , torrential^34^. All measurements were reviewed by two independent cardiologists that were dedicated to echocardiographic evaluation.

### Clinical follow-up

The primary endpoint was a composite outcome, consisting of all-cause mortality and hospitalization due to worsening HF within one year after TTVR. As secondary endpoints, all-cause mortality, cardiovascular mortality, and hospitalization due to worsening HF within one year after TTVR were examined separately. All suspected adverse events were independently adjudicated by the local heart team according to the criteria of the Valve Academic Research Consortium 2^35^. The need for hospitalization due to worsening HF was determined based on the attending physicians’ discretion, without any prespecified criteria. The occurrence of clinical events was recorded from the admission records and outpatient medical records. In addition, HF medication, including beta-blockers, renin-angiotensin system (RAS) inhibitors, and aldosterone antagonists, or dosage with a standardized furosemide equivalent were recorded at baseline^36^.

### Statistical analysis

Continuous variables were tested for normal distribution using the Kolmogorov–Smirnov test. Normally distributed variables are presented as the mean ± standard deviation and compared using t-tests. In contrast, non-normally distributed variables were expressed as medians with an interquartile range (IQR) and compared between groups using the Mann–Whitney U-test. Categorical data were presented as numbers and percentages, and the differences between groups were evaluated using the chi-square test. Logistic regression analysis was performed to detect parameters that were related to the MELD-XI score and those that were related to the reduction of MELD-XI score. The variables with *p* < 0.05 in the univariate analysis were incorporated into a multivariable regression model. The receiver-operating characteristic (ROC) analysis was used to investigate the cut-off value of the MELD-XI score, to predict the composite outcome within one year after TTVR. According to the cut-off value, patients were categorized into two groups: high MELD-XI and low MELD-XI. In addition, Harrell’s C-statistic was used to compare the predictive ability of hepatorenal functional markers for the composite outcome by the area-under-the-curve analysis, accounting for censoring. Kaplan–Meier cumulative event curves for the composite outcome, all-cause mortality, cardiovascular mortality, and HF hospitalization were generated by using two groups according to the high or low MELD-XI score. Differences between the groups were compared using the log-rank test. Univariate and multivariable Cox-proportional hazard models were used to calculate the hazard ratios (HRs) with 95% confidence intervals (CIs) for the MELD-XI score for the composite outcome within one year after TTVR. In a univariate analysis, we analyzed the HRs of clinical parameters (model 1) and cardiac and procedural parameters (model 2) that were determined, considering the number of end points and multicollinearity. In the multivariable analyses, covariates were included that showed significance (*p* < 0.05) in the univariate analyses. Statistical significance was set at *p* < 0.05. All analyses were conducted using Stata 15.1 (StataCorp, College Station, TX, USA) or JMP version 14.0 for Mac (SAS Institute Inc., Cary, NC, USA).

### Ethical statement

Our registry was approved by the local Ethical Committee at the University of Bonn in accordance with the Declaration of Helsinki. Informed consent was obtained from all participants after receiving a full written and oral explanation of the purpose of our registry.

## Supplementary Information

Supplementary Information.
