# Supplementary material for: Prognostic impact of hepatorenal function in patients undergoing transcatheter tricuspid valve repair
Source: Sci Rep. 2021 Jul 13;11:14420. doi: 10.1038/s41598-021-93952-9 (PMC8277825; doi:10.1038/s41598-021-93952-9)
Supplement: Supplementary file 1 — Supplementary Information. [file 41598_2021_93952_MOESM1_ESM.docx]

**Prognostic Impact of Hepatorenal Function in Patients Undergoing**

**Transcatheter Tricuspid Valve Repair**

**Supplemental materials**

Running title: MELD-XI score and TTVR Prognosis

Tetsu Tanaka, MD*; Refik Kavsur, MD*; Atsushi Sugiura, MD, PhD;

Johanna Vogelhuber, MD; Can Öztürk, MD; Marcel Weber, MD; Vedat Tiyerili, MD; Sebastian Zimmer, MD; Georg Nickenig, MD; Marc Ulrich Becher, MD

*Affiliations:*

^1^ Heart Center Bonn, Department of Medicine II, University Hospital Bonn, Germany

* contributed equally to this paper

**Supplemental Figure 1. The ROC curve analysis for one-year composite outcome after TTVR**

**
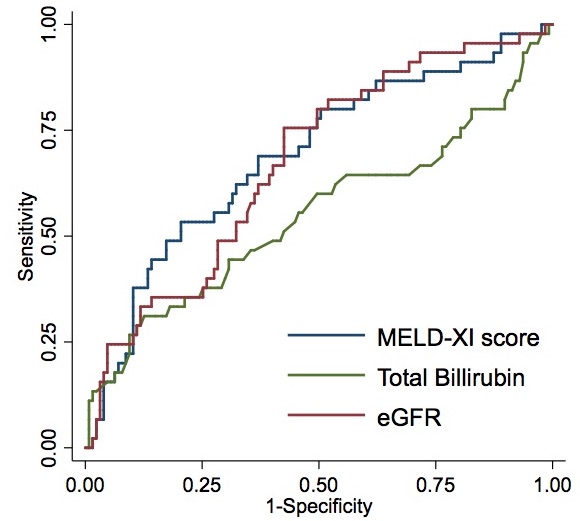
**

The ROC curve analysis of the MELD-XI score (AUC: 0.69; p=0.0009), total bilirubin (AUC: 0.55; p=0.09), and eGFR (AUC: 0.67; p=0.004) for the composite outcome within one year after TMVR.

AUC = area under the curve

**Supplemental Table 1. Linear regression analysis to predict MELD-XI score**

|  | Univariate analysis | | | Multivariable analysis | | |
| --- | --- | --- | --- | --- | --- | --- |
|  | β coefficient | 95%CI | p value | β coefficient | 95%CI | p value |
| Age (years) | 0.18 | 0.03 to 0.33 | 0.02 | 0.32 | 0.17 to 0.46 | <0.0001 |
| Male | 0.25 | 0.10 to 0.40 | 0.001 | -0.08 | -0.05 to 0.24 | 0.29 |
| LVEF (%) | -0.33 | -0.47 to -0.18 | <0.0001 | -0.29 | -0.49 to -0.17 | 0.001 |
| LVEDV (ml) | 0.26 | 0.11 to 0.41 | 0.001 | 0.12 | -0.07 to 0.25 | 0.15 |
| SPAP (mmHg) | -0.07 | -0.23 to 0.10 | 0.42 |  |  |  |
| TAPSE (mm) | -0.12 | -0.28 to 0.03 | 0.12 |  |  |  |
| RV diameter (mm) | 0.18 | 0.03 to 0.33 | 0.02 | 0.12 | -0.02 to 0.27 | 0.10 |
| RA area (mm^2^) | 0.14 | -0.02 to 0.29 | 0.09 |  |  |  |
| TR severity: 4+ or 5+ | 0.02 | -0.13 to 0.17 | 0.76 |  |  |  |
| Legends: MELD-XI score = Model for End-stage Liver Disease eXcluding International normalized score; LVEF = left ventricular ejection fraction; LVEDV = left ventricular end-diastolic volume; SPAP = systolic pulmonary artery pressure; TAPSE = tricuspid annular plane systolic excursion; RV = right ventricle; RA = right atrium; TR = tricuspid regurgitation. | | | | | | |

**Supplemental Table 2. The Harrell’s C-statistics for one-year composite outcome after TTVR**

|  | C-statistics | 95%CI | p value |
| --- | --- | --- | --- |
| MELD-XI score | 0.68 | 0.60 - 0.76 | <0.0001 |
| eGFR (ml/min/1.73m^2^) | 0.66 | 0.46 - 0.66 | <0.0001 |
| Total bilirubin (mg/dl) | 0.56 | 0.58 - 0.74 | <0.0001 |
| Legends: MELD-XI score = Model for End-stage Liver Disease eXcluding International normalized score, eGFR = estimated glomerular filtration rate. | | | |

**Supplemental Table 3. Univariate analysis for predictors of the one-year composite outcome after TTVR**

|  | HR | 95% CI | p value |
| --- | --- | --- | --- |
| Model 1: Clinical parameters | | | |
| Age (year) | 1.01 | 0.97 - 1.05 | 0.77 |
| Male | 1.61 | 0.89 - 2.90 | 0.12 |
| BMI (kg/mm^2^) | 0.96 | 0.90 - 1.02 | 0.22 |
| Diabetes mellitus | 0.98 | 0.49 - 1.82 | 0.94 |
| Hypertension | 1.79 | 0.65 - 7.40 | 0.29 |
| COPD | 1.51 | 0.78 - 2.78 | 0.21 |
| Prior MI | 0.72 | 0.34 - 1.39 | 0.34 |
| Prior CABG | 1.52 | 0.77 - 2.82 | 0.22 |
| Prior Valve intervention | 0.79 | 0.41 - 1.45 | 0.45 |
| Atrial fibrillation | 0.76 | 0.31 - 2.53 | 0.61 |
| Trans-tricuspid lead | 1.69 | 0.93 - 3.04 | 0.08 |
| Model 2: Echocardiographic parameters | | | |
| LVEDV (ml) | 1.07 | 0.99 - 1.15 | 0.09 |
| SPAP (mmHg) | 0.99 | 0.97 - 1.02 | 0.61 |
| RA area (mm^2^) | 1.01 | 0.98 - 0.99 | 0.62 |
| RV diameter (mm) | 1.00 | 0.97 - 1.03 | 0.96 |
| TR severity: 4+ or 5+ | 1.28 | 0.71 - 2.32 | 0.41 |
| EROA (mm^2^) | 0.99 | 0.98 - 1.01 | 0.39 |
| Vena contracta (mm) | 1.01 | 0.94 - 1.09 | 0.73 |
| Edge-to-edge repair | 1.49 | 0.73 - 3.45 | 0.29 |
| Post TVPG (mmHg) | 1.07 | 0.81 - 1.38 | 0.63 |
| Legends: MELD-XI score = Model for End-stage Liver Disease eXcluding International normalized score, BMI = body mass index; COPD = chronic obstructive pulmonary disease; MI = myocardial infarction; CABG = coronary artery bypass graft; LVEDV = left ventricular end-diastolic volume; PAP = systolic pulmonary artery pressure; RA = right atrium; RV = right ventricle; TR = tricuspid regurgitation; EROA = effective regurgitant orifice area; LA = left atrium; TVPG = tricuspid valve pressure gradient. | | | |

**Supplemental Table 4. Serial assessment of hepatorenal function at baseline and six-month follow-up after TTVR (n=63)**

| Parameters | |
| --- | --- |
| MELD-XI score | |
| Baseline | 10.8 ± 5.0 |
| Follow-up | 11.0 ± 7.4 |
| MELD-XI reduction | 26 (41.2) |
| eGFR (ml/min/1.73m^2^) |  |
| Baseline | 42.6 [30.9, 59.3] |
| Follow-up | 44.8 [27.3, 58.0] |
| Total bilirubin (mg/dl) | |
| Baseline | 0.70 [0.47, 0.89] |
| Follow-up | 0.62 [0.38, 0.98] |
| Legends: MELD-XI score = Model for End-stage Liver Disease eXcluding International normalized score, eGFR = estimated glomerular filtration rate. | |

**Supplemental Table 5. Logistic regression analysis for the MELD-XI reduction at six-month follow-up (n=63)**

|  | Univariate analysis | | | Multivariable analysis | | |
| --- | --- | --- | --- | --- | --- | --- |
|  | OR | 95% CI | p value | OR | 95% CI | p value |
| Postprocedural TR <3+ | 3.19 | 1.08 - 9.41 | 0.04 | 3.37 | 1.09 - 10.40 | 0.03 |
| LVEDV (per 10 ml increase) | 0.85 | 0.72 – 0.99 | 0.04 | 0.98 | 0.95 - 0.99 | 0.03 |
| High MELD-XI score (≥14) | 0.38 | 0.11 – 1.35 | 0.13 |  |  |  |
| Age (years) | 1.01 | 0.94 - 1.08 | 0.87 |  |  |  |
| Male | 0.44 | 0.14 - 1.35 | 0.15 |  |  |  |
| NYHA class IV | 1.55 | 0.44 - 5.18 | 0.5 |  |  |  |
| Atrial fibrillation | 3.03 | 0.32 - 28.81 | 0.33 |  |  |  |
| Lead across tricuspid valve | 0.98 | 0.34 - 2.80 | 0.97 |  |  |  |
| LVEF (per 10% increase) | 1.11 | 0.73 - 1.69 | 0.61 |  |  |  |
| TAPSE (mm) | 1.06 | 0.96 - 1.18 | 0.24 |  |  |  |
| RV diameter (mm) | 0.99 | 0.94 - 1.04 | 0.71 |  |  |  |
| RA area (mm^2^) | 0.99 | 0.94 - 1.04 | 0.73 |  |  |  |
| Post mean TVPG (mmHg) | 0.76 | 0.46 - 1.27 | 0.26 |  |  |  |
| TR reduction of grade one at least | 1.48 | 0.65 - 7.98 | 0.95 |  |  |  |
| Legends: MELD-XI score = Model for End-stage Liver Disease eXcluding International normalized score, TR = tricuspid regurgitation; NYHA = New York Heart Association; LVEF = left ventricular ejection fraction; LVEDV = left ventricular end-diastolic volume; TAPSE = tricuspid annular plane systolic excursion; RV = right ventricle; RA = right atrium; TVPG = tricuspid valve pressure gradient. | | | | | | |
